# Climate Change Enhanced Carotenoid Pro-Vitamin A Levels of Selected Plantain Cultivars

**DOI:** 10.3390/plants9040541

**Published:** 2020-04-22

**Authors:** Beloved Mensah Dzomeku, Julian P. Wald, Jens Norbert Wünsche, Donatus Nohr, Hans K. Biesalski

**Affiliations:** 1 CSIR-Crops Research Institute, P.O. Box 3785, Kumasi AK000-AK911, Ghana; 2 Institute of Nutrition Science (140a), University of Hohenheim, Garben strasse 30, D-70593 Stuttgart, Germany; julian.p.wald@googlemail.com (J.P.W.); Donatus.nohr@uni-hohenheim.de (D.N.); Hans-K.Biesalski@uni-hohenheim.de (H.K.B.); 3 Department of Crop Science, Crop Physiology of Specialty Crops (340f), University of Hohenheim, 70599 Stuttgart, Germany; jnwuensche@uni-hohenheim.de

**Keywords:** UV-B radiation, micronutrient, carotenoids, climate variability, Musa spp

## Abstract

Diet diversification and the exploitation of traditional, micronutrient-rich germplasm of staple crops are generally regarded as sustainable and low-cost approaches to increase the micronutrient intake of resource-poor people. Sun’s UV index was collected daily throughout the year. The study assessed the seasonality of provitamin A carotenoids in three plantain cultivars in response to climatic condition. Fruits were harvested at three maturities and freeze-dried before analysis. The results showed that there were high levels of the sun’s UV-B radiations throughout the year with the highest occurring from November to May when the area experienced clear skies with minimal cloud cover. These high levels of the sun’s UV-B index occurred between 9.00 h GMT and 17.00 h GMT. The study also showed that α-carotene content increased with maturity in “Apantu” during the rainy seasons ranging from 95 to 172 μg/100 g of dry pulp. Similar trends were observed during the dry season with a range of 28 to 489 μg/100 g. The α-carotene contents were very high in the periods of high sun’s UV-B radiations compared to the periods of low sun’s UV-B radiations. The α-carotene levels in the giant French plantains showed similar trends. Intermediate French “Oniaba” and False Horn “Apantu” plantain cultivar showed the highest content of β-carotene during the dry season. The high provitamin A carotenoid levels in the cultivars coincided with the high levels of the sun’s UV index.

## 1. Introduction

Diet diversification and the exploitation of traditional, micronutrient-rich germplasm of staple crops are generally regarded as sustainable and low-cost approaches to increase the micronutrient intake of resource-poor people [1]. Micronutrient deficiencies were reported to reach clinical levels in many countries in sub-Saharan Africa (Grieger and Clifton, [2]; Black, et al., [3], including Ghana [4,5]. In most of these countries, plantains are a major staple crop, often grown in association with other food crops, like cassava, sweet potato and vegetables.

Micronutrient deficiencies are known to afflict millions of people in the developing world. Studies have shown that diet rich in carotenoids is associated with reduced risk of heart disease and cancer [1,2,3,6]. Though micronutrients are readily available in fruits, vegetables and animal products, they are often not easily accessible to the poor. Often, the micronutrient-rich food products are undervalued by those who need them most. The diets of these vulnerable consist mainly of carbohydrates such as roots and tubers and cereals. 

Reports from WHO/World bank indicate that around two billion people worldwide suffer from micronutrient malnutrition [7]. Vitamin A deficiency (VAD) is reported to be the leading cause of preventable blindness in children and increases the risk of disease and death from severe infections. In pregnant women VAD causes night blindness and may increase the risk of maternal mortality [5,8]. Vitamin A (vit A) deficiency is reported to be a public health problem in 118 countries, affecting an estimated 250 million preschool children [8]. Up to 500,000 children become blind every year, and 50% die within 1 year of becoming blind [9]. Reports indicate that close to 20 million pregnant women are also vitamin A vit A deficient. Iron (Fe) deficiency is reported to be the most common micronutrient deficiency in the world [9]. The WHO estimates that up to 1/3 of the world’s population is Fe deficient [10,11,12]. In addition, close to 60% of the population in developing countries is thought to also be at risk of Zinc (Zn) deficiency [9]. Micronutrient deficiency in developing countries therefore is alarming; though all the crops rich in these nutrients abound [13]. Several indigenous vegetables are very rich in micronutrients compared to the introduced varieties and fruits in these countries; however, their low consumption is mostly due to stigmatization. It is often as a result of the mindset and the lack of information on the nutritional values of the crops. 

Reducing vitamin and mineral deficiencies is an essential part of the overall effort to fight “hidden hunger” and malnutrition. Supplementing micronutrients in the form of pills or syrups, fortifying processed foods with micronutrients and breeding crops for increased micronutrients levels are three commonly applied options to increase micronutrients intake [14]. However, to reach resource-poor people, who often have limited access to health-care systems and/or formal markets, diet diversification and the exploitation of traditional, micronutrient-rich germplasm of staple crops are generally regarded as more sustainable and low-cost approaches, that fit well in traditional food systems [1,15]. 

It is reported that certain vitamin concentrations in some fruits and vegetables may be affected by irradiation; however, there is naturally a large variation of these vitamins in fruits and vegetables. These variations are also dependent on the plant cultivar, growing conditions, maturity of the edible portion, post-harvest handling and storage conditions [16].

Studies have demonstrated that there is a high level of variation in both carotene and vitamin C content in tropical fruits, with the greatest variation coming from cultivar type [17,18]. Growing season, location and harvest time also influence nutrient composition of fruits [19]. 

Plantains (*Musa* spp) are among the most popular starchy staple grown in the humid tropics where micronutrient deficiency is prevalent. It comprises the world’s fourth most important food crop, with an annual production of about 100 million tonnes. They constitute a starchy staple across some of the poorest parts of the world, including sub-Saharan Africa (with per capita consumption up to 400 kg) [20]. Plantains are a good source of income for millions of rural households in vulnerable developing countries with about 25 million tonnes produced annually [21]. Various cultivars of plantain are consumed at the green or half-ripe stages as cooked starchy carbohydrate or when ripe as dessert banana. For millions of rural poor in West Africa, plantains are not only a primary source of energy but also an important source of dietary minerals and vitamins. As such, the promotion and increased production of micronutrient-rich cultivars (Shetty, [1] has the potential to have a significant long-term beneficial impact on the incidence of micronutrient deficiencies. 

The occurrence of orange-fleshed Musa cultivars with exceptionally high provit A carotenoids (pVACs) contents has been described (Englberger, et al., [22,23,24,25,26] and results of large-scale screening activities showed that there is a high degree of genetic variability in the fruit pVACs contents of Musa genotypes, with values ranging from 0 to as high as 11,337 μg/gdw [27]. Plantains, a major staple in West African countries, seem to have higher pVACs contents than dessert bananas. However, even within the plantain subgroup, substantial variation exists, suggesting that gains could be achieved by promoting more pVACs-rich cultivars over traditionally consumed cultivars. An ex-ante impact assessment using household data from plantain-growing regions of Ghana indicated that substituting Musa cultivars with high-pVACs content could reduce the burden of vitamin A deficiency-related illness by up to 17% and be more cost effective than other health-nutrition interventions [28,29]. 

Several studies have reported the nutritional composition of plantains [30,31,32]. Nutritional Analysis of within-fruit, within-hand and within-plant as well as the between-plant of plantains from West and Central Africa showed that significant variations exist in both provitamin A carotenoids (pVACs) and mineral micronutrient (Fe, Zn) contents across all sample groups [33].

In this era of climate change there are speculations of its effect on crop nutrition and nutrient composition in crops. Several studies have shown climate change and its effect on micronutrients in crops; and other studies have also reported an increase in CO_2_ concentration in the atmosphere could alter the levels of proteins, B vitamins and zinc [34,35]. Nutrient reduction is projected to be particularly severer in sub-Saharan Africa, where levels of undernutrition are already higher and diets are more vulnerable to direct impacts of climatic parameters associated with climate change.

Climate change is known to impact on the accumulation of minerals and proteins in crops, with elevated CO_2_ being the underlying factor of most of the reported changes. These reports indicated that the effects are dependent on the type, intensity and duration of the imposed stress, plant genotype and developmental stages. Strong interactions (both positive and negative) are known to be found between individual climatic factors and soil available nitrogen (N), potassium (K), iron (Fe) and phosphorus (P). Some authors have proposed that future interventions to ensure plentiful, safe and nutritious food for the world’s population may need to rely on breeding for nutrients under the context of climate change, including legumes in cropping system, better farm management practices and utilization of microbial inoculants that enhance nutrient availability [36]. Nevertheless, evidence shows that under seminatural field conditions UV-B radiation is not as detrimental for plant growth and physiology, as previously believed [37]. Furthermore, UV-B radiation effects are species specific and depend on interactions with other environmental parameters [38,39,40]. Studies under controlled environment showed that some species exposure to UV radiation had increased carotenoid concentration, in others there was a reduction. However, the effect of UV radiation on plant is also reported to be influenced by life forms [41]. Several studies on the effect of UV radiation on carotenoids in plants species showed that its effects could be variety dependent [42,43,44] (Reeds et al., [42]; Barnes et al., [43]; Correia et al., [44] as each plant species behaves differently to adapt to the environment. 

This study was therefore conducted to assess the pro-vitamin A active carotenoid levels of three selected plantain cultivars with season and maturity.

## 2. Materials and Methods

Weather data were collected from flowering to harvest using AccuWeather application using Huawei phone collaborated with Davis Vantage Pro2 Plus weather station. Weather data were collected daily at hourly intervals throughout the study period. Weather data (temperature, relative humidity and sun’s UV index) were taken hourly from 9.00 Hours GMT to 17.00 Hours GMT for two conservative years (2014–2015).

Fruit samples were collected from a plantain orchard of the Crops Research Institute at Fumesua, Kumasi. The experimental design was Randomised Complete Block (RCBD) with four replications. The plant spacing was 3 m × 2 m between and within rows giving plant population of 1667 plants per hectare. Each plot contained 25 plants. Planting was staggered (April and September) for fruiting to coincide with the rainy (June to October) and dry (November to May) seasons. At bolting, plants were tagged and monitored. Field maintenance was carried out by slashing as and when needed and no pesticide application was carried out. No chemical fertilizer was applied to the fields. However, pruning of dried leaves was done regularly.

Three plantain cultivars—”Apantu” (False horn), “Apem” (Giant French) and “Oniaba” (medium French)—were tagged at flowering in the Crops Research Institute plantain orchard at Fumesua, Kumasi. Two bunches from each plot were harvested from the middle portion of each plot at each maturity date (70, 80 and 90 days after flowering). This was done for dry and rainy seasons. These were pooled together and three bunches were randomly selected and sent to the International Sweetpotato center (CIP) laboratory for freeze-drying and milling. In the laboratory, samples for freeze drying were taken from the second and third hands. Four fingers were taken, peeled and sliced longitudinally for freeze drying. Samples were milled after drying and packaged in black polyethylene bags and stored at −20 °C for five days before shipment. Milled samples were carried in black polyethylene bags and sent to Institut für Biologische Chemie und Ernährungswissenschaft, University of Hohenheim, Germany for provitamin A analysis. 

## 3. Carotenoid Analysis

Samples were extracted and analyzed as previously described Wald et al. [45]; however, minor adaptations in the extraction process as well as in the chromatographic settings were made. Briefly, about 100 mg sample were extracted in 2 mL tubes using a mixture of methanol and hexane (containing 1 mmol BHA and BHT as well as internal standard: apocarotenal; 30 min at room temperatures). Phase separation was enhanced using saturated sodium chloride solution and centrifugation (1 min, 13,200 rpm). The nonpolar layer was removed and stored separately. The remaining suspension was washed twice with hexane, thus pooling the organic phases that were evaporated and disolved in isopropanol, subsequently. After membrane filtration (PTFE, 0.20 µm, 13 mm), solutions were injected into the Shimadzu HPLC system (CMB-20A communication module, SIL-20AC HT autosampler, LC-20AT liquid chromatography pumps, SPD-M20A diode array detector). For chromatographic separation, a Prontosil 200-3-C30 column (Bischoff) was used, however, flow rate was reduced to 1.5 mL/min that led to gradient adaptations (min/%A: 0/100; 18/40; 22/0; 25/0). Provitamin A active carotenoids were detected using PDA (450 nm). Data were analysed using analysis of variance (ANOVA).

## 4. Results and Discussion

### Carotenoid Contents Depending on Maturation and Seasonality

The sun UV radiation index was studied for two consecutive years using AccuWeather. The study showed that the sun’s UV radiations were higher from November to May, which coincided with the dry and beginning of the rainy seasons. (Table 1). The sun’s UV radiation index was relatively lower during the rainy seasons (June to October) (Table 1). The period of November to April is characterized by clear atmosphere with strong sunlight. The average minimum and maximum temperatures during dry season and rainy seasons range between 27 and 39 °C and 25 and 28 °C respectively. The relative humidity during the dry periods were between 25% and 35% whereas the wet periods experienced relative humidity of between 75% and 85%. 

**Figure 1:**
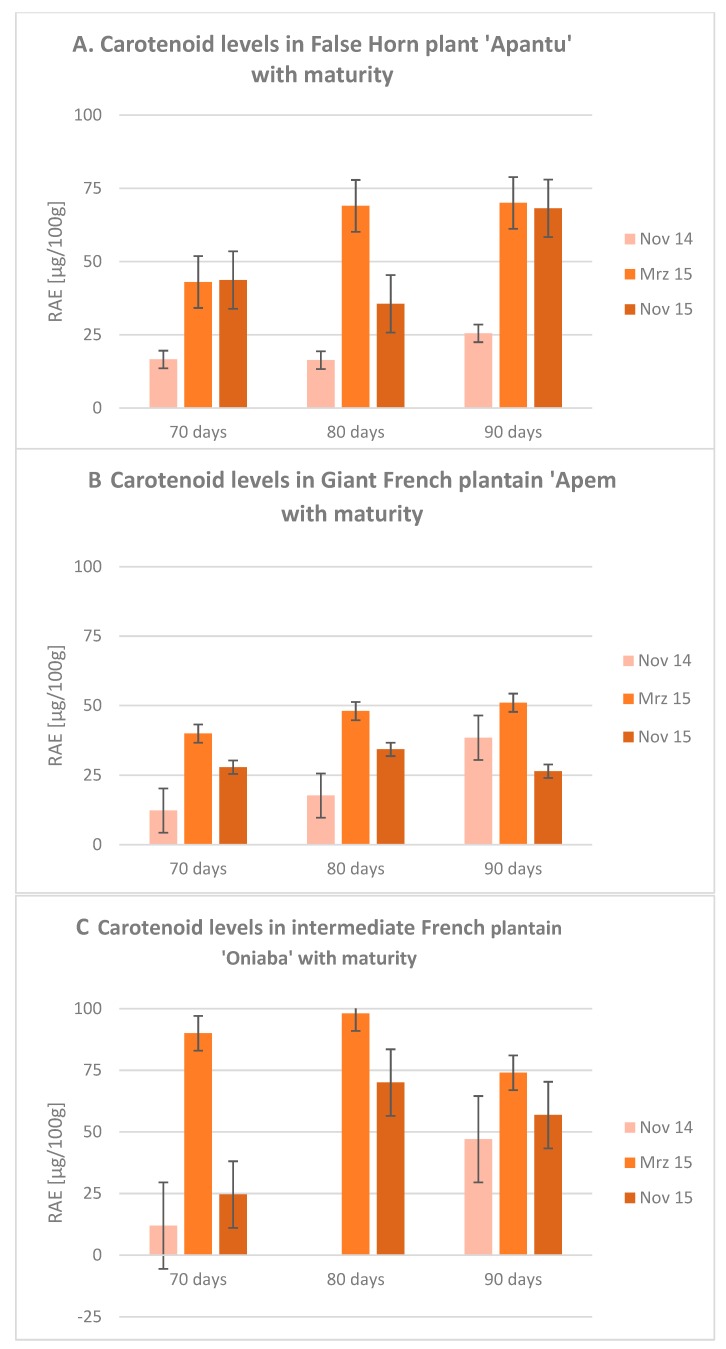
Maturation and season dependent changes in Retinol Activity Equivalents (conversion factors: 1:12 for β-carotene, 1:24 for other provitamin A active carotenoids) in three different plantain cultivars, (**A**) “Apantu”, (**B**) “Apem” and (**C**) “Oniaba”. Plantain samples were harvested after 70, 80 and 90 days after flowering in dry and rainy seasons.

Provitamin A active carotenoids in three different plantain cultivars were determined (Table 2, Table 3 and Table 4). Whereas cryptoxanthin was not detected in any of the plantain samples, substantial amounts of α- and β-carotenes were observed. Intermediate French “Oniaba” was the plantain cultivar with the highest content of provitamin A investigated in this study, followed by False Horn “Apantu” and Giant French “Apem” respectively (Figure 1). Despite minor deviations, provitamin A contents in each plantain cultivar increased with higher degree of maturity. Our study revealed that periods of high levels of the sun’s UV-B index coincided with high levels of provitamin carotenoids in plantain (Table 1 and Table 4). The seasonality of the levels of carotenoid could be attributed to sun’s UV radiation index having a significant impact on the enhanced contents. The results further showed that β-carotene content was higher than α-carotene in the False Horn “Apantu” cultivar (Table 4). However, the trend was different in the Giant French “Apem” and the intermediate French “Oniaba”. Overall, there were significant differences (*p* < 0.05) in carotenoid levels across cultivars and maturities. The results corroborated well with that of Shen et al., [46], Mark and Tevini, [47] and Sullivan and Teramuta, [38]. In their study, Shen et al. [46] showed an 8% increase in leaf carotenoid content even at low UV-B exposure (+9.75 μW/cm^2^) from 2 to 8 days exposure, compared to nonexposed plants. However, at high UV-B exposure (+20.76 μW/cm^2^), the carotenoid content increased rapidly after a day’s exposure (10.41% higher than the control). In a similar study on tomato fruits exposed to UV-B, Pérez, et al., [48] observed that exposure of tomato fruits to UV-B before harvest resulted in accumulation of lycopene and β-carotene content. They also observed that the highest accumulation of lycopene and β-carotene was induced by a UV-B dosage of 0.075 Wh m^−2^ after 22 h of exposure. It was concluded that carotenoids synthesis was promoted by moderate UV-B radiation before harvest, nevertheless, the time and duration of exposure were paramount to a plant compound-specific response. Hu, et al.; [49] showed that LED and UV irradiations significantly accelerated ripening in orange and also caused changes in the soluble sugar, organic acid and carotenoid contents. Unlike fruit subjected to dark shade (DS) treatment, UV-treated (UVA, UVB, and UVC) fruits experienced significant increase in total soluble sugar, fructose and glucose contents.

Intermediate French plantain “Oniaba” showed higher levels of α-carotene than β-carotene at early fruit filling stages in the dry season and reduced level at physiologically matured stages (Table 3). This period of reduction also coincided with the lower level of the sun’s UV index in the field (Table 1). Studies have shown a wide inter- and intraspecific differences in response to UV-B irradiation with respect to growth, production of dry matter and physiological and biochemical changes in plants [50,51,52]. This phenomenon was also observed by Lidon and Ramalho [53] when leaves of rice were exposed to UV-B stress, the chlorophyll apparatus was observed to increase; however there was recovery 14 days after the stress. Studies showed that UV irradiation influenced accelerated orange ripening and also caused significant changes in the soluble sugar, organic acid and carotenoid content [49]. While in some species exposure to UV radiation could increase carotenoid concentration, in others there may be a reduction. However, the effect of UV radiation on plant physiological responses is also reported to be influenced by life forms [41]. It is also recorded to be influenced by abiotic factors like air temperature (Mark and Tevini, [47], atmospheric carbon dioxide concentrations (Sullivan [54] and soil nitrogen (Hunt and McNeil [55]; Correia et al., [56] and moisture content [38,57]. The high carotenoid levels in our study also coincided with the periods of high ambient temperature levels. 

The study further showed that α-carotene content increased with maturity in “Apantu” (Table 3) during the rainy season ranging from 95 to 172 μg/100 g of dry pulp. Similar trends were observed during the dry season with a range of 28 to 489 μg/100 g. The α-carotene contents were very high in the minor rainy and dry seasons compared to the major rainy season. The α-carotene levels in the French plantains showed similar trends (Table 3). 

β-carotene levels were also observed to be low in the major rainy season and very high during the minor rainy and dry seasons when the sun’s UV radiations were high. The carotenoid levels were also high with maturity. The major rainy season is often between March and July and the minor season between September and November each year. One major characteristic of the major rainy season is thick cloud cover with reduced sunlight. The minor season is associated with clear atmosphere with strong sunlight and intermittent rains.

It is believed that plantains may be responding to high levels of sun’s UV-B, either by stimulating protection mechanisms or by activating repair mechanisms to cope with the different types of stress. Plantains are known to contain high levels of phenolic compounds which are commonly used by plants in response to UV-B and also to attenuate the penetration of the UV-B range of the sun’s spectrum into deeper layers of the tissue. 

These high α-carotene levels with seasonality could be attributable to the high incidence of the sun’s UV index. It was observed that during the time of the study, high levels of the sun’s UV indices were observed (Table 1 and Table 2). Sun’s UV index were observed between 9.00 GMT and 17.00 h GMT daily from November to May.

The results confirm the assertions of Lu et al. [17]; Dhuique-Mayer et al. [18] and González-Molina et al. [19]; Lee and Kader, [58] that the nutrient composition of tropical fruits are influenced by growing season, location and harvest time of fruits. This could be good news for addressing micronutrient deficiency in developing countries as food-based strategy; however, the challenge could be the bioavailability of the carotenoids and their conversion to retinol for use by the body [59]. Climatic conditions including light and average temperature have a strong influence on the chemical composition of horticultural crops [60]. It is reported that phenolic compounds in fruits are influenced by UV-B [61,62,63,64,65,66]. However, the plantain plant has laticifers containing latex full of phenolic compounds within the leaves and fruits that could be potential adaptation mechanism for high solar UV radiations. Studies showed that UV-B radiation had a positive effect on the flavour of melons; carotenoid content slightly increased in older leaves in plants in response to UV-B radiation [67]. In a study of exposing *Malva parviflora* L., *Plantago major* L., *Rumex vesicarius* L. and *Sismbrium erysimoids* to two weeks of enhanced UV.B radiation, the carotenoid levels were increased [68]. While previous studies recorded substantial variability of fruit carotenoid content in Musa spp. suggesting possibilities for breeding, our studies have added a new dimension of sun’s UV-B, being that it influences the provitamin content of plantain pulp. In their study it was observed that carotenoids are precursors for norisoprenoid compounds in grapes (Razungles et al., [69], hence the suggestion that UV-radiation may also affect grape and wine flavour. Understanding the mechanism(s) by which physiological processes are damaged, repaired and/or protected is therefore important for elucidating the eco-physiological role of UV-B radiation in enhancing carotenoid levels in fruits.

## 5. Conclusion

UV-B radiation continues to be relevant in plant physiology especially with the impact of enhanced UV-B in sunlight resulting from stratospheric ozone depletion. The current high incidence of the sun’s UV-B and its effects on secondary metabolites in food crops calls for concern. Our results shows that increases in the sun’s UV-B can cause photomorphogenic as well as genetic and physiological changes in plants. The study revealed that provitamin A carotenoids content of plantain cultivars vary with seasonality and with maturity. The seasonality of the UV-B radiation from the sun should trigger new research approaches of interactions between abiotic and biotic stresses and physiological responses of plants, especially metabolites. Variation in the carotenoid levels in plantain could be influenced by sun’s UV-B index. Climate change with its accompanying complexities therefore could have a positive effect on some plant secondary metabolites and improve nutritional content of some crops. 

## Figures and Tables

**Table 1:** Monthly Means values of sun’s UV-B Index for 2014 and 2015.

Months												
Year	Jan	Feb	Mar	Apr	May	Jun	Jul	Aug	Sept	Oct	Nov	Dec
2014	12 ± 0.1	12 ± 0.1	12 ± 0.01	12 ± 0.1	12 ± 0.1	7 ± 0.01	7 ± 0.01	8 ± 0.01	8 ± 0.01	8 ± 0.01	9 ± 0.01	12 ± 0.01
2015	12 ± 1.0	12 ± 0.1	12 ± 0.02	12 ± 0.1	12 ± 0.1	7 ± 0.01	7 ± 0.01	7 ± 0.01	8 ± 0.01	8 ± 0.01	9 ± 0.01	12 ± 0.01

**Table 2:** Provitamin A active carotenoid contents of three different plantain cultivars at maturation and seasonality.

Cultivar	Maturity (Days) during the Rainy Season I					
Cultivar	α-carotene Levels µg/100 g Edible Pulp) at Three Maturities			β-carotene Levels µg/100 g Edible Pulp) at Three Maturities		
	70	80	90	70	80	90
“Apantu”	95.7 ± 9.11a	108 ± 13.8a	171 ± 32.2a	151 + 15.7a	142 + 9.66	220 ± 29.2a
“Apem”	78.6 ± 5.34b	128 ± 4.31b	291 ± 7.43b	108 ± 9.3b	148 + 9.25	386 ± 4.09b
“Oniaba”	83.7 ± 12.4ab	-	328 ± 57.7c	102 ± 22.1b	-	400 ± 86.4b
CV	12.4	10.4	30.6	10.7	11.8	30.1
LSD (*p* < 0.05)	12.0	13.2	20.2	8.9	10.9	12.3

**Table 3:** Provitamin A active carotenoid levels µg/100 g edible pulp) three different plantain cultivars at maturation and seasonality.

Cultivar	Maturity (Days) During Rainy Season 11					
	α-carotene Contents at Three Maturities			β-carotene Content at Three Maturities		
	70	80	90	70	80	90
“Apantu”	297 ± 38.1a	254 ± 36.3a	474 ± 45.0a	362 ± 20.7a	288 ± 29.4a	567 ± 59.7a
“Apem”	310 ± 3.26b	356 ± 5.79b	379 ± 2.12b	221 ± 42.6b	279 ± 39.3a	210 ± 44.5b
“Oniaba”	160 ± 9.77c	533 ± 60.4c	435 ± 4.70c	209 ± 11.8b	558 ± 65.4b	451 ± 3.89c
CV	15.1	20.4	40.6	10.7	11.8	45.1
LSD (*p* < 0.05)	9.3	15.2	12.2	12.9	11.9	15.3

**Table 4:** Provitamin A active carotenoid levels µg/100 g edible pulp of three different plantain cultivars at maturation and seasonality.

Maturity Date (Days) during the Dry Season						
Cultivar	α-carotene Contents at Three Maturities			β-carotene Content at Three Maturities		
	70	80	90	70	80	90
“Apantu”	283 ± 5.08a	420 ± 2.80a	489 ± 7.57a	358 ± 5.24a	577 ± 4.09a	573 ± 7.79a
“Apem”	310 ± 3.26a	356 ± 5.79b	379 ± 2.12b	299 ± 2.31b	371 ± 5.09b	386 ± 4.09b
“Oniaba”	719 ± 6.93b	793 ± 8.39c	542 ± 0.36c	685 ± 7.63c	744 ± 10.3c	577 ± 2.75a
CV	18.1	20.4	40.6	10.7	31.8	50.1
LSD (*p* < 0.05)	30.3	15.2	12.2	12.9	21.9	16.3
